# Is the mediating effect of psychosocial stress on the income–health relationship moderated by income inequality?

**DOI:** 10.1016/j.ssmph.2022.101302

**Published:** 2022-11-29

**Authors:** Sigbjørn Svalestuen

**Affiliations:** Department of Social Sciences, UiT The Arctic University of Norway, Tromsø, Norway

**Keywords:** Income inequality, Income, Health, Psychosocial stress, Material deprivation, Multilevel moderated mediation

## Abstract

**Background:**

There now exists a rich body of literature on the relationship between income, income inequality, and health. The discussion about the impact of income and income inequality on health includes psychosocial mechanisms, such as long-term perceptions of inferiority and social positioning, material advantage from income, and the structural conditions that define what people can do with their material resources.

**Aims:**

This study investigated the extent to which income's effects on health are mediated by psychosocial stress, and to what extent those effects are moderated by country-level income inequality and economic development.

**Methods:**

Data were collected from The European Social Survey, round 7. Multilevel moderated mediation analysis was applied to estimate the extent of psychosocial stress mediation of the effects of income on self-rated health. Moderated parameters were estimated over country-level income inequality and economic development.

**Results:**

Significant full or partial meditation by psychosocial stress was found in all 20 countries studied. Effects moderated by income inequality and GDP per capita showed expected relationships but failed to reach conventional levels of statistical significance.

**Conclusions:**

Individual-level income remains important for explaining the income–health gradient in self-rated health in Europe. The income–health relationship and the extent to which it is mediated by psychosocial stress varies among countries but is not significantly moderated by contextual income or income inequality. Policies should be aimed at allowing a greater proportion of people to live in material comfort and reduced sense of financial precarity, and protecting individuals from harmful consequences of low income.

## Highlights

• Multilevel moderated mediation can uncover whether direct or mediated effects of income co-vary with country-level variables.
• The effect of income on self-rated health is mediated by psychosocial stress in all countries under study.
• Neither the mediated nor direct effect of income is moderated by income inequality or GDP per capita.

## 1 Introduction

Income has long been of interest to health and health inequality researchers. Studies have suggested that health gaps tend to be wider between individuals in the lower to middle parts of income distribution. It has also been shown that the annual life expectancy is increasing for the highest income quartile, while for the lowest income quartile, life expectancy has stagnated (Kinge et al., 2019). Moreover, the income–health gradient for self-rated health is steeper than the education–health and occupation–health gradients (Olsen et al., 2020). The nonlinear relationship between income and self-rated health suggests that whatever mechanisms explain these inequalities, their effects are stronger among those living on a very low income (Mackenbach, 2019; Mackenbach et al., 2005). While empirical evidence for the income–health gradient is well documented, authors disagree on causal mechanisms; that is whether the income–health gradient is socially determined (Gravelle, 1998; Lynch et al., 2000; Wilkinson, 1999), that ill-health generates income inequality through health selection (O’Donnell, Doorslaer, & Van Ourti, 2015; García-Gómez, 2011), or that the flow of causation is bi-directional over the life-course (Hoffmann et al., 2018; Rehnberg et al., 2021).

Further disagreements on the causal processes linking income to health can be made within the social determinants of health perspective. Materialists argue that the aggregate relationship between income and population health is an artifact of the individual level income–health gradient (Gravelle, 1998). Neo-materialists (Lynch et al. 2000, 2004) argue that income at both the individual and society levels fundamentally reflect the detrimental effects of living in poor material conditions combined with politico-economic processes that govern private resources and public welfare systems. Psychosocial stress theorists (Marmot, 2001; Pickett & Wilkinson, 2015; Wilkinson, 1999) argue that income inequality itself is the issue; relative positioning in the income hierarchy can generate long-term biological stress responses with detrimental health effects.

This study applied multilevel mediation modeling to investigate the effects of individual income on self-rated health. It examined the extent to which psychosocial stress mediates those effects. Also examined was the extent to which the direct effect of income and the mediated effect of psychosocial stress are moderated by country-level income and income inequality.

## 2 Income, income inequality, and health

Studies on the effect of income inequality on health can be traced back to 1979. Rodgers (1979) conducted a cross-sectional international analysis on the association between the Gini coefficient and national mortality statistics. At the individual level, it is widely accepted that higher incomes and other socioeconomic characteristics are associated with many indicators of health (Lynch et al., 2004; O’Donnell et al., 2015). This association presents the shape of a gradient in even the wealthiest of countries (Olsen et al., 2020).

However, the empirical relationship between income inequality and population health is contested. Two important reviews (Lynch et al., 2004; Wilkinson & Pickett, 2006) published in the early 2000s serve as useful illustrations. Lynch et al. (2004) concluded that income inequality in affluent countries is not associated with population health differences as a general phenomenon. It was cited that most of the negative or mixed findings were conducted post-1995, presumably using better quality data. Some studies were characterized as showing mixed results due to findings that were inconsistent between population age groups and a priori predictions of the income inequality hypothesis (IIH). The researchers also noted that multilevel studies found no significant associations after controlling for within-country individual factors and sensitivity to country inclusion in the sample. They found stronger evidence for the IIH in studies using aggregate regional and state-level data from the United States. Again, multilevel studies presented less support. Furthermore, both aggregate and multilevel evidence suggested little or no effect of income inequality in a number of other rich countries.

Wilkinson and Pickett (2006) concluded that 70% of the analyzed papers were wholly supportive of the IIH. The researchers argued that null findings can primarily be explained by the size and type of the analyzed area; analyses of parishes, counties, and census tracts tended to yield unsupportive findings more frequently than country and regional data. Further, results were sensitive to control variable selection. While their perspective generally related to the psychosocial links between income inequality and health outcomes, they did not ignore material factors in their analysis. They argued that the social structure is built substantially on material foundations. The core of the argument is thus that materialism matters, but the link between income inequality and health is not completely explained by material factors. The psychosocial perspective they proposed provides a new path to health outcomes through the negative effects of social comparison.

Wagstaff and Van Doorslaer (2000) explained the divergent findings to some extent. They argued that data from aggregate-level studies are insufficient for discriminating between competing hypotheses. They reviewed evidence for the absolute-income hypothesis, the relative-income hypothesis, the IIH, and the deprivation hypothesis. Out of the four, they found strong support only for the absolute-income hypothesis. They concluded that income inequality only affects population health due to its effects on the poor. They found no convincing support of the relative-income hypothesis whatsoever. They further noted that eight out of nine hypotheses will predict an association between average health and income inequality. Observing this effect empirically will not distinguish between the proposed explanations for the prediction. The same is true for average income with the same eight hypotheses. They concluded that research on these hypotheses up to the 2000s had been incapable of shedding any light on relative income and income inequality affecting individual health. Moreover, the individual studies that were feasibly able to do so showed less than compelling results for the relative-income hypothesis and the IIH.

Beckfield (2004) found that the relationship between health and inequality disappeared in fixed-effects models that addressed unobserved heterogeneity. Mellor and Milyo (2002) argued that previous findings of an association between income inequality and health are partly the product of an ecological fallacy and the failure to control for individual covariates, year effects, and geographic characteristics. Kragten and Rözer (2017) found that while OLS and multilevel models yielded a positive association between income inequality and health, fixed-effects models and analyses of sub-groups associated income inequality with poor health. Torre and Myrskylä (2014) found increases in age- and gender-specific mortality rates where there were increases in income inequality even when controlling for shared period factors and country fixed effects. The strongest effects were observed for children and young-to-middle-aged men. Curran and Mahutga (2018) applied fixed-effects modeling to compare differential effects of income inequality between countries with varying levels of economic development. The results showed a larger effect of income inequality in poorer countries. Similarly, Oorschot (2013) found that while the IIH was supported in low- and middle-income countries, there was no significant relationship between life expectancy and income inequality in high-income countries. They argued that, to some extent, a high level of economic development tempers the potential negative effects of income inequality due to the population's command over essential public goods and services (and more of them). However, they also found that the relationship between levels of income inequality and life expectancy was not robust over time. They also found that the level of economic development moderated the effects of the level of wealth on life expectancy.

Doorslaer and Koolman (2004) found that income contributed to health inequality. However, there were significant variations between European countries in how much health inequality could reasonably be attributed to income differentials. While they found that health inequality was positively correlated with income inequality per se, it was a weaker link than in previous research. Gugushvili et al. (2020) found that perceived changes in income inequality affected self-reported health, as opposed to a direct effect of income inequality. Their work expanded on the psychosocial mechanism because they concerned themselves with how people see and feel inequality in their everyday lives. McFarland, Hill and Montez (2022) found that the association between income inequality and life expectancy in the United States was moderated by state-level policy liberalism. Layte et al. (2019), using data from five cohort studies from four European countries, found higher levels of inflammation and greater differentials in inflammation by socioeconomic positioning in countries with comparatively high levels of income inequality.

In a meta-analysis, Ngamaba et al. (2018) found that subjective well-being and income inequality were only significantly associated in developing countries. Maynou et al. (2015) investigated spatiotemporal processes of regional health convergence and found that convergence rates varied significantly. A recent panel data analysis of 26 European countries for the period 1995 to 2004 found no evidence of a relationship between life expectancy at birth and income inequality (Blázquez-Fernández et al., 2018). Olstad et al. (2022) compared the extent to which psychosocial stress mediates the effect of subjective social status, perceived income adequacy, and educational attainment on self-rated overall health between four countries at varying levels of income inequality. They found no evidence for psychosocial stress being a more important mediator of the association between subjective social status and self-rated overall health in more unequal societies.

One systematic review concluded that area-level income inequality was associated with poorer mental health Tibber et al., 2022 in spite of several methodological limitations in the studies. Sommet et al. (2018) found that income inequality and psychological health are linked, but only for people experiencing financial scarcity. Further, in a systematic review of income inequality and depression, Patel et al. (2018) found that around two-thirds of the 26 reviewed studies supported a link between income inequality and risk of depression.

Pickett and Wilkinson (2015) re-reviewed the literature with explicit consideration given to the potential causal relationship between income inequality and health. They found that the body of evidence to date indicated a strong causal connection due to satisfying the major epidemiological criteria for causality: temporality, plausibility, consistency, and a lack of alternative explanations. Further, they argued that null findings can be explained by inappropriate scales of measurement, mediating variables being used as controls/confounders, use of subjective measurements of health, and short follow-up periods. While their review did not explicitly address the causal mechanisms (focusing instead on methodological criteria for evaluating cause-and-effect), they persisted in the most parsimonious explanation for these effects being social class accentuation and status differentiation. They noted that future studies should make explicit attempts to clarify the causal nature of the empirical relationship.

Another review evaluated the research by distinguishing research efforts that were based on longitudinal, panel, and cross-sectional data (Truesdale & Jencks, 2016). Overall, the only relatively strong relationship identified was between income inequality and social inequalities in life expectancy in single country time series. This suggests that the relationship is weak in cross-sectional and panel data analyses. The evidence for a relationship between average life expectancy and income inequality were considered weak in time series and panel data evidence and is merely moderate in the cross-sectional context.

Findings on the empirical relationship between income inequality and health are mixed. Diverging conclusions can be explained in part by the methodology used (e.g., criteria for support/no-support) and differences in framing (e.g., “evidence for a causal claim” and “averages and disparities”). However, these reviews show that the effects and theoretical pathways of income inequality on health are still under discussion more than 40 years after Rodgers (1979).

### 2.1 Psychosocial stress and environment: mechanisms

Wilkinson (1994) argued that as societies progress through epidemiological transitions—shifting from infectious diseases as the main causes of death to degenerative cardiovascular diseases and cancers—the mechanisms explaining income gradients in health transition as well. Within-country income gradients in mortality remained, but gross domestic/national product (GDP/GNP) per capita as a predictor of between-country mortality underperformed as explanans in states with long life expectancies. Rather, country-level income inequality showed a more robust association with life expectancy in wealthier countries. Although the impact of psychosocial factors on health had previously been discussed, Wilkinson expanded and suggested that health outcomes are “less a matter of the immediate physical effects of inferior material conditions than of the social meanings attached to those conditions and how people feel about their circumstances and about themselves.” Proponents of the relative deprivation argument cite the fact that there is an income gradient in health outcomes rather than a difference explained by poverty alone. They also note that mortality disadvantages remain even with rising real incomes and that living standards among the poorest are much higher than before.

Early formulations of psychosocial theory argued that the social environment could alter host susceptibility to pathogenic agents by affecting neuroendocrine function (Cassel, 1976). Future studies carried these ideas forward, as psychosocial frameworks typically direct attention to endogenous biological responses to human interactions (Krieger, 2001). Long-term feelings of subordination or inferiority are expected to stimulate chronic stress responses that have consequences for physical and mental health (Bambra, 2011). Psychosocial variables like feelings of control, anxiety, insecurity, depression, and social affiliation have been cited as successfully explaining the health gradient. These stimulations may have an effect on health either directly or indirectly. Directly could be through the influence of social relations on neuroendocrine pathways to disease (such as chronic stress leading to wear and tear on the body and mind; allostatic load), and indirectly through stress-related behaviors, such as smoking (Marmot, 2001; Wilkinson, 1994, 1999).

The theoretical perspectives of psychosocial stress emphasize social integration. Inequality produces disintegration and individualism, which undermine the potential beneficial health effects of social support. This links the psychosocial stress hypothesis to the concept of social capital (Putnam, 2000). Also linked is the notion that generalized trust and social cohesion are conditions for a number of factors associated with well-functioning societies (Uslaner & Mitchell, 2005). Social capital, cohesion, and trust generate social support through friendships and social networks. This effect has been argued to be as protective for health as smoking is deleterious (Pickett & Wilkinson, 2015). However, where there is great inequality, there also tends to be underinvestment in the various forms of soft capital, such as education and medical services. Overlapping with Wilkinson empirically and theoretically, these factors have typically been used in materialist arguments (Beckfield, 2004).

### 2.2 Neo-materialism: mechanisms

The psychosocial environment as the missing link for explaining the non-relationship between GDP per capita and mortality in high-income countries was criticized by Lynch et al. (2000). They argued that the selection of high-income countries was too restrictive and found a stronger relationship when the sample size was extended to include countries outside of the OECD. More importantly, they disagreed about the underlying mechanisms linking income inequality to mortality statistics. They argued that income inequality does not reflect feelings of inferiority and the perception of place in a social hierarchy based on relative position according to income. Instead, they stated that income inequality is one of many manifestations of historical, cultural, and political-economic processes that influence the private resources available to individuals and shapes the nature of public infrastructure. While the psychosocial environment hypothesis assumes universal associations (due to persistent perceptions of relative position regardless of actual living conditions), the neo-materialist view assumes contextual processes. The criticism is partially based on the practical implications of dealing with health inequality under psychosocial theories and goes so far as to argue that the psychosocial environment hypothesis implies mass psychotherapy to alter perceptions of relative disadvantage. Neo-materialist explanations argue that the income–health gradient exists because of a combination of the material possibilities of individual income and the conditions that govern what income enables. Despite the redistributional and decommodifying efforts of the welfare state through cash transfers, taxation, and benefits, there still exist substantial inequalities in material advantage across the globe (Mackenbach, 2012). Income gives access to goods and services and limits exposures to physical and psychosocial risk factors. Neo-materialism gives primacy to structure when explaining health outcomes and health inequality. Individual agency is limited, and public policy and services create the pattern of social inequality (Bambra, 2011).

### 2.3 Expectations

Psychosocial stress is understood as one possible pathway at the individual level by which income may impact health (Kawachi et al., 2002; Wilkinson, 1999). Income may affect health more directly if material conditions are strained (Gravelle, 1998; Lynch et al., 2000). Psychosocial stress may fully or partially mediate the effect of income on health, leading to the following expectations:

• **H1:***Psychosocial stress significantly mediates the relationship between individual income and health outcomes.*
• **H2:***Income has a significant direct effect on health outcomes at the individual level.*

The IIH assumes that large income differences intensify social hierarchies and class conflict, as well as increase feelings of relative deprivation (Elgar, 2010), thus intensifying the effect of the “status syndrome”. Further, material conditions are expected to worsen overall in the countries with low economic development:

• **H3:***The mediating effect of psychosocial stress and the direct effect of income are significantly moderated by income inequality.*
• **H4:***The mediating effect of psychosocial stress and the direct effect of income are significantly moderated by economic development.*

## 3 Statistical analysis

As the classic mediation model (Baron & Kenny, 1986) assumes independent observations, multilevel mediation analysis should be applied in contexts of clustered data to account for bias in standard errors due to a lack of independence in observations (Tofighi & Thoemmes, 2014). This is the case for the European Social Survey (ESS). Two hypotheses assume that the mediated and direct effects from the multilevel mediation model are moderated by country-level income inequality (**H3**) or economic development (**H4**). The 1-1-1 multilevel mediation framework is therefore extended by including country-level moderators to predict random (income) slopes (Tofighi et al., 2013). This is achieved by including interaction terms between the moderator, treatment, and mediating variables. Once the base models are fitted, different levels of the moderator at which effects will be calculated are set by the researcher (Tingley et al., 2014). Coefficients and 95% bootstrap confidence intervals are calculated for mean and one standard deviation in levels of income inequality and economic development, respectively.

**Figure B1:**
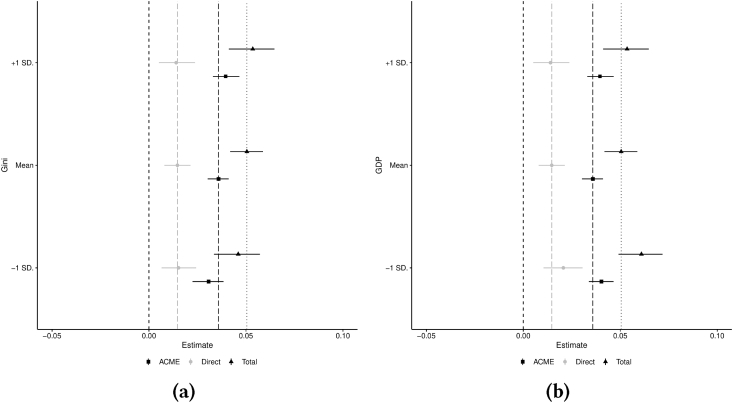
Left: ACME, direct, and total effects from multilevel moderated mediation model at three different levels of income inequality. Right: ACME, direct, and total effects from multilevel moderated mediation model at three different levels of GDP. Both figures include 95% bootstrap confidence intervals. Vertical lines are centered on the mean estimate and zero. Both models based on *N* = 28814 observations by listwise deletion.

Missing values were addressed by multiple imputation using the expectation-maximization with bootstrapping (EMB) algorithm using the Amelia package (Honaker et al., 2011). Final results were combined over separate estimations from *m* = 5 imputed datasets. Household income data were unavailable from Estonia. Estonia was therefore omitted from the final sample. Results from models using listwise deletion are available in figure B1a and B1b in the appendix. Base multilevel models were fit using the lme4 package (Bates, 2010). Moderated mediation analysis based on lmer objects were fit using the mediation package (Tingley et al., 2014). All analyses were conducted in R.

## 4 Data

Individual level variables were collected from the seventh round of the ESS (ESS, 2014). This round was selected because it is the only round to date containing a module on social inequalities in health in Europe.

Self-rated health was measured using the single item “How is your health in general? Would you say it is …” completed on a five-point scale with answers ranging from “very bad” to “very good.”. Self-rated health has been applied in health and health inequality research both as a single item measurement (Beckfield et al., 2013; DeSalvo et al., 2006; Lorem et al., 2020) and a multi-item composite indicator (Olsen et al., 2020). Self-rated health has been shown to predict other health outcomes such as mortality risk (Lorem et al., 2020). Self-rated health was selected because it reflects interlinked social, psychological, and biological processes (Balaj, 2020) and should be an responsive indicator to perceptions of ones position in the income gradient and the potential effect of income inequality.

The ESS measures income by giving respondents a showcard with ten income brackets in the local currency and ask respondents to place their households total net income in one of the brackets. While the categories on the scorecard are intended to represent household income deciles, deviations from the expected uniform distribution in many countries warrants some caution in interpreting the income measure as such. Rather, the income measurement should be interpreted as an individuals position on their countries socioeconomic ladder (Donnelly & Pop-Eleches, 2018).

Marmot and Wilkinson (2001) define feelings of control, anxiety, insecurity, depression, and social affiliation as psychosocial indicators. The ESS7 contains a selection of items related to these dimensions, of which 14 items were selected for constructing the index. An overview of the components is available in Table 1. Insecurity and feelings of control were captured by indicators of autonomy at work and feelings about the household income. Depression and stress-related symptoms were captured by indicators of happiness and sadness, self-reported depression, sleep quality, and feelings of lethargy. Social affiliation was captured using indicators of how often a respondent meets friends and participates in social activities, self-reported number of intimate relationships, and feelings of loneliness.

**Table 1:** Summary statistics prior to EMB imputation. Calculated scale reliability psychosocial stress index: *α* = 0.785. See appendix for complete component transformation scheme. Estonia (*N*_*j*_ = 2045) was dropped prior to EMB imputation as household income data were unavailable, yielding a final sample of *N* = 38140 in *j* = 20 countries post imputation.

Variables	Mean	Std. Dev.	Min.	Max	N	NA
**Individual data**						
Self-rated health	2.82	0.92	0	4	40136	49
Income	5.32	2.78	1	10	31889	8296
Psychosocial stress	0.95	0.41	0	3	34372	5813
Age	49.28	18.74	14	114	40086	99
Gender	0.53	0.50	0	1	40163	22
Education	12.90	3.94	0	50	39828	357
Partner	0.59	0.49	0	1	40035	150
**Country data**						
Gini index	0.30	0.04	0.25	0.38	20	0
Top 10% income share	0.35	0.05	0.29	0.50	20	0
Top 1% income share	0.11	0.03	0.07	0.17	20	0
Bottom 50% income share	0.21	.03	.13	.25	20	0
GDP per capita	41007.75	11696.99	25297.95	66018.42	20	0
**Index components**						
Feelings about income	0.95	0.84	0	3	39809	376
Autonomy at work	1.18	0.93	0	3	36595	3590
Influence work policy	1.64	1.01	0	3	36401	3784
Depression, how often	0.44	0.67	0	3	39975	210
Effort, how often	0.65	0.78	0	3	39964	221
Happy, how often	1.04	0.81	0	3	39812	373
Enjoying life, how often	1.06	0.85	0	3	39851	334
Feel sad, how often	0.53	0.67	0	3	39933	252
Can't get going, how often	0.55	0.71	0	3	39882	303
Sleep was restless	0.77	0.84	0	3	40007	178
Meet friends often	1.11	1.05	0	3	39595	590
Intimate relationships	1.65	0.64	0	3	39835	350
Social activities, how often	1.48	0.74	0	3	39603	582
Lonely, how often	0.39	0.69	0	3	39940	245

The majority of items were measured using a four-point scale ranging from “None or almost none of the time” to “All or almost all of the time”. Autonomy at work and influence over work policy were measured on an eleven-point scale. Respondents were provided seven-point scales to determine how often they meet friends and their number of intimate relationships. A five-point scale distinguished their frequency in social activities as compared to others. These items were collapsed to comply with the four-point scale applied in all other items. Items were inverted where necessary to conform to low-to-high directionality in the psychosocial stress measurement prior to final calculation. Finally, the psychosocial stress index was created using the arithmetic mean, giving all items equal weight. A complete schematic of component transformation is available in table C1 in the appendix.

Education is often used as a measurement of socioeconomic status alongside income (Olsen et al., 2020). However, education is also an important determinant of income (Lahelma, 2001) and research has suggested some reporting heterogeneity in self-rated health between educational groups (Balaj, 2020). There are theorized mechanisms linking education to health through alternate pathways; such as individual cognition or early-life socioeconomic circumstances (Lindberg et al., 2022). Controlling for education serves to parse this variance from the income indicator.

Co-habitation with a partner was included as the income indicator measures household as opposed to individual income. Controlling for co-habitation with a partner thus serves to partial out the income differentials reported from combined incomes.

Gender was included as a control, as gender differences in the proportion of people reporting poor or very poor health in the ESS7 have been observed (Balaj et al., 2017). Age was included as a control as the income–health gradient and its mechanisms may vary over different stages of the life course (Hoffmann et al., 2018; O’Donnell et al., 2015; Rehnberg et al., 2021).

Country-level indicators were collected from the Quality of Government standard dataset (Teorell et al., 2021) and the World Inequality Database. Country level income is measured as GDP per capita. Income inequality is captured by the Gini coefficient in the main model. Following De Maio and Fernando (2007) and Pickett and Wilkinson (2015), top-and-bottom sensitive income inequality indicators were included for sensitivity purposes. Summary statistics are provided in Table 1.

## 5 Results

**Fig. 1:**
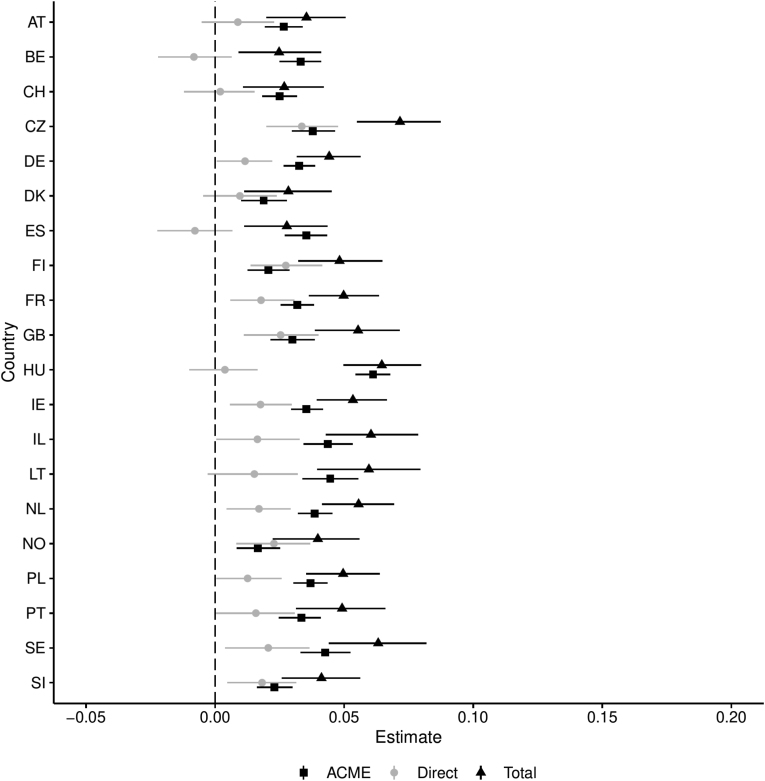
Overview of mediated, direct, and total effect sizes including 95% bootstrap confidence intervals, by country. Effects were controlled for age, gender, education, and living with a partner. Final results combined over separate results from *m* = 5 imputed datasets.

**Fig. 2:**
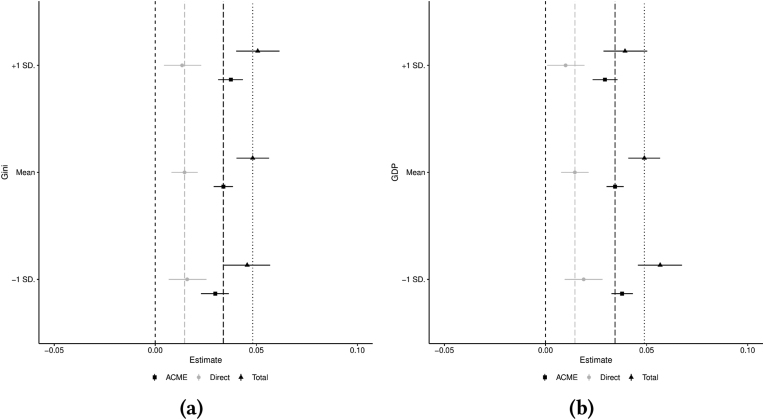
*Left: ACME, direct, and total effects from multilevel moderated mediation model at three different levels of income inequality. Right: ACME, direct, and total effects from multilevel moderated mediation model at three different levels of GDP. Both figures include 95% bootstrap confidence intervals. Vertical lines are centered on the mean estimate and zero. Final results combined over separate results fromm* = 5 *imputed datasets.*

**Fig. 3:**
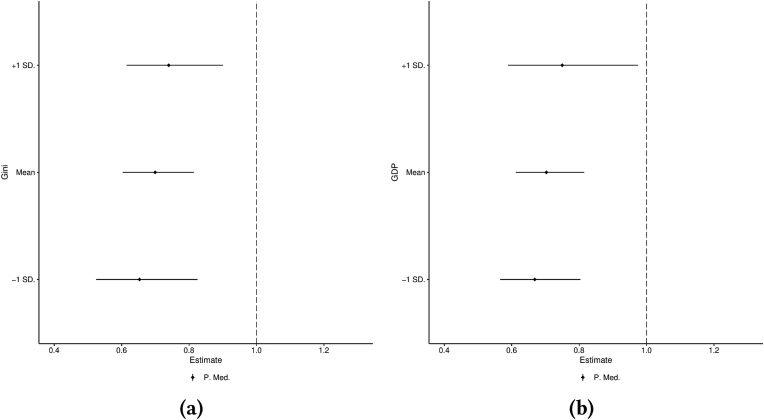
*Left: Proportion of mediated effect of income from multilevel moderated mediation model at three different levels of income inequality. Right: Proportion of mediated effect of income from multilevel mediation model at three different levels of GDP* per capita*. Both figures include 95% bootstrap confidence intervals.*

Results from multilevel mediation modeling are presented by country in Table 2 and Fig. 1. Results from moderated mediation models are presented in Fig. 2, Fig. 3. Results from models using the top 10%, top 1%, and bottom 50% income share as indicators for income inequality are available in appendix A.

**Table 2:** Overview of effects by country. Effects were controlled for age, gender, education, and living with a partner. Total sample size post EMB imputation *N* = 38134. Final results combined over separate results from *m* = 5 imputed datasets.

Country	ACME	Direct	Total	P. Med.	N
Austria	0.025	0.008	0.032	0.768	1795
Belgium	0.032	−0.008	0.025	1.297	1769
Switzerland	0.025	0.002	0.027	0.932	1532
Czech Republic	0.039	0.034	0.073	0.534	2148
Germany	0.033	0.011	0.044	0.749	3045
Denmark	0.019	0.012	0.031	0.619	1502
Spain	0.035	−0.007	0.028	1.275	1925
Finland	0.020	0.029	0.049	0.412	2087
France	0.031	0.019	0.050	0.621	1917
Great Britain	0.031	0.022	0.052	0.586	2264
Hungary	0.061	0.002	0.063	0.966	1698
Ireland	0.036	0.016	0.052	0.698	2390
Israel	0.045	0.011	0.056	0.811	2562
Lithuania	0.045	0.013	0.058	0.777	2250
Netherlands	0.039	0.016	0.056	0.704	1919
Norway	0.017	0.021	0.038	0.447	1436
Poland	0.038	0.011	0.049	0.771	1615
Portugal	0.034	0.015	0.049	0.689	1265
Sweden	0.041	0.021	0.062	0.662	1791
Slovenia	0.022	0.017	0.039	0.558	1224

The average causal mediation effect (ACME) of psychosocial stress varied from 0.017 (Norway) to 0.061 (Hungary). The mediating effect of psychosocial stress on the income–health relationship is clearly significant in all countries. The specific mediation process only becomes clear in relation to the size and significance of the direct effect of income. Income's direct effects ranged from the smallest of −.008 (Belgium) to the largest of 0.034 (Czech Republic). There was evidence for two different mediation processes. In countries where the direct effect of income is significant (Czech Republic, Finland, France, Great Britain, Ireland, Netherlands, Norway, Portugal, Sweden, and Slovenia), the total effect of income was partially mediated by psychosocial stress. The direct effect of income is not significant in Austria, Belgium, Switzerland, Germany, Denmark, Spain, Hungary, Israel, Lithuania, and Poland. In these countries, the evidence suggested full psychosocial mediation. Spain and Belgium showed marginally different patterns to the other countries. Point estimates suggested competitive mediation, which is a negative direct effect of income competing with a positively mediated effect of psychosocial stress. However, as the direct effect of income in these countries are non-significant, full mediation is concluded.

Total effects varied in line with each component effect. In countries with an insignificant direct effect of income, the total effect was slightly greater than the mediated effect of psychosocial stress (excepting Spain and Belgium). In countries with a significant direct effect of income, the component effects tended to have similar proportions.

Consulting the “P. Med” column in Table 2, the proportion of the total effect mediated by psychosocial stress varied from 41.2% to 96.6%, excluding proportions above 1. This table shows the problematic nature of evaluating the proportion of the mediated effect in isolation; competitive mediation means that the proportion of the total effect being mediated is uninterpretable as a traditional proportion.

Psychosocial stress accounted for a substantial proportion of the total effect of income in all countries studied, showing support for hypothesis **H1**. Hypothesis **H2** found support in countries with partial mediation, amounting to 10 out of 20 countries included in the sample.

### 5.1 Relationship with income inequality and GDP per capita

Fig. 2a plots the mediated, direct, and total effects over three levels of income inequality. Point estimates of the mediated effect of psychosocial stress on the income-health gradient are similar across the income inequality scale. The 95% bootstrapped confidence intervals suggest that the differences in the mediated effect of psychosocial stress between income inequality levels is not significant. The same can be said of the total and direct effect of income on self-rated health. Both the direct and mediated effects are significantly different from zero, supporting hypotheses **H1** and **H2**.

Fig. 2b plots the mediated, direct, and total effects over three levels of income measured in GDP per capita. Contrary to income inequality, the point estimates in the direct effect are the main drivers of changes in the total effect of income. At a higher GDP, the direct effect of income tends to be smaller, while the mediated effect stays relatively stagnant over different levels of economic development. However, neither the direct effect of income, the mediated effect of psychosocial stress, or total effect are significantly affected by the level of economic development.

Fig. 3a and b plot the proportion of the total effect being mediated by psychosocial stress at three different levels of income inequality and GDP per capita, respectively. These figures are extremely similar but for different reasons. Increases in the point estimate of the mediated effect account for most of the variation in the total effect over income inequality levels. Conversely, decreases in the direct effect account for most of the variation in the total effect over different levels of GDP per capita. In both cases, this results in a minor increase in the predicted proportion of mediated effect. Similar to previous estimates, however, the proportion mediated effect does not vary significantly at different levels of either income inequality or economic development. Any differences between the observed effects failed to reach any conventional measure of statistical significance. **H3** and **H4** are not supported.

## 6 Discussion

Individual income matters for self-rated health, regardless of country-level income and income inequality. This does not mean that material poverty is the only factor in play. The psychosocial stress mediator accounted for 69.3% (median) of the total effect of income on self-rated health across countries, suggesting that psychosocial stress is correlated with income and self-rated health and accounts for a substantial amount of the covariance between income and self-rated health.

The IIH argues that long-term feelings of inferiority act as primary mechanisms of why income matters for health (Pickett & Wilkinson, 2015; Wilkinson, 1994) and assumes that the situation worsens in societies with higher levels of income inequality. That is, the potential for feeling worse is a result of relatively lower positioning in the hierarchy, exacerbated by the gulfs in income generated by income inequality. This prediction largely failed in the context of ESS data.

The more probable reason for a strong mediating effect is rooted in the lived experience of stress and how this covaries with individual-level incomes. Rather than considering the psychosocial environment a standalone effect resulting in stress, psychosocial stress may result from low income itself. This would be due to those in low-income groups having a greater prevalence of less comfort, more worries about finances, depression, fatalistic tendencies, lack of control, and lacking social affiliation.

Lynch et al. (2000) argued that “health inequalities result from differential accumulation of exposures and experiences that have their sources in the material world.” They also posited that the income distribution is a result of historical, cultural, and politico-economic processes that shape the nature of public infrastructure. The psychosocial interpretation argues that while the negative emotive experience is rooted in material income conditions, the negative effects occur due to a low position on the social hierarchy, specifically (Wilkinson, 1994, 1999). Kawachi et al. (2002) argued that, in reality, these explanations are not mutually exclusive or possible to disentangle. One key factor when discussing psychosocial and material causation is distinguishing between underlying pathways to health, and initial causes of health. Psychosocial factors like low social status and lack of control are often labeled psychosocial determinants, although they may be triggered by material factors. It is here that the theories intersect most notably, when considering how material hardship in lower socioeconomic groups is a likely source of psychosocial stress (Mackenbach, 2012). Empirical overlap between material factors and the hypothesized emotional experiences of inequality is likely. Following Kawachi et al. (2002), if we consider the psychosocial stress hypothesis to be a causal pathway, there is no apparent conflict between the two. All material resources have some psychosocial meaning attached to it, but they also provide a sense of material security. This sense of material security (or scarcity) combined with both material and neo-material perspectives would help explain why there is a gradient in the first place. The end result is less focus on a position of inferiority and the subjective experience of income inequality, and instead, more focus on the psychological benefits of financial stability and security.

This implies that psychosocial pathways are not an initial cause. The lack of an appreciable effect of income inequality alone on effect sizes suggests as much. Pickett and Wilkinson (2015) argued that because income inequality has been linked to lower levels of social cohesion and generalized trust, it means that inequality must act as a social stressor. The psychosocial explanation of the income effect is argued to be biologically plausible when linked with the detrimental health effects of chronic stress. What is missing empirically is the expected exacerbation of mediated and total effects over income inequality. The psychosocial stress effect of income is therefore to a greater extent about general feelings or behaviors associated with low income. The effect of income being fully mediated by the psychosocial stress index in many countries suggests that psychosocial stress matters for self-rated health, linking material goods to psychosocial pathways.

The IIH, regardless of mechanisms or empirical support, cannot exist without an income–health gradient. One can apply most theoretical frameworks and reach similar conclusions that there are statistically appreciable differences in health based on income groups. Theoretical divergence on this effect occurs because of the shape of that gradient. That is, income–health differences are not merely the differences in health between the rich and the poor. An income gradient in health is the necessary backbone upon which a hypothetical income inequality effect rests. The IIH is motivated by the inability of the income–health hypothesis to explain relative homogeneity in population health between the wealthiest of countries (Marmot & Wilkinson, 2001). In other words, the IIH exists only in relation to the income–health hypothesis. Regardless of the effect or lack thereof from income inequality on health, the literature mostly agrees on policy recommendations: reducing income inequality will lead to better population health. Reducing income inequality strategically means raising disadvantaged people out of material hardship, falling back on the established mechanisms of the income–health hypothesis.

### 6.1 Strengths and weaknesses

A major strength of this study is its novelty. Several studies have embraced the comparative nature of the IIH (Layte et al., 2019; Olstad et al., 2022), but no study to date has tested the IIH in a multilevel moderated mediation framework. Further, the study establishes a novel psychosocial stress measurement based on the conceptual framework presented by Marmot (2001) that may be used or amended for future comparative studies on the income–health gradient and tests of the IIH.

The psychosocial stress index includes items measuring depressive symptoms, lethargy, and restless sleep. Single-item stress measurements have been shown previously to converge on similar psychological symptoms, sleep disturbance items, and well-being (Elo, Leppänen, & Jahkola, 2003). While depression is also a component of health in self-ratings, self-rated health as a concept is comprehensive, inclusive, and non-specific. It applies contextual frameworks of evaluation to ones own health status such as culturally varying conceptions of health, makes reference to previous experiences and the health status of others, and reflects cultural conventions in expressing health and health related issues (Jylhä, 2009). As long as psychosocial stress is partially defined by depression, anxiety, and the like, some conceptual overlap between health and psychosocial stress is inevitable. However, correlations in the ESS7 show that items in the index reflecting depression and well-being are more strongly correlated internally than with self-rated health. The psychosocial stress index only accounts for *R*^2^ = 0.21% of the variance in self-rated health. These points suggest that psychosocial stress and self-rated health are related, but distinct concepts.

As noted by Beckfield (2004), sample (country) variations may impact the estimated country-level correlations. While there are ample sample sizes at the individual level, a small number of countries means comparatively large standard errors and increases the probability of sub-sample variability. Further, the sample does not fully reflect the global variation in income inequality or economic development. While this region is theoretically relevant for the IIH, future studies should aim to include a larger sample of countries that represent the global variation in income inequality and economic development.

Zhao, Lynch Jr., and Chen (2010) argued that partial mediation suggests an incomplete theoretical framework, but notes that there are instances where the direct effect is an a priori expectation. While it can be argued that material effects themselves should be mediated, by, for instance, measuring house ownership or similar sources of capital, the direct effect is simply assumed to represent material effects of income. There are at least two behavioral mechanisms that may bias this interpretation of a direct effect as materialistic: scarcity theory, (Mullainathan & Shafir, 2013) where an additional cognitive load due to poverty means individuals prioritize short-term needs at the expense of long-term planning and decision-making; and diffusions of innovations (Rogers, 1962), which is the tendency for the rich or highly educated to adopt innovative health behaviors early. Effectively estimating potential biasing effects of scarcity theory necessitates a measurement of an individual's cognitive capacity and their relative cognitive load specifically attributable to scarcity. That is not exactly a standard indicator in international comparative survey data. Additionally, the diffusion of innovations mechanisms are interrelated with other theoretical assumptions and difficult to parse from existing frameworks. The adoption of healthy behaviors and health-related technology could proxy this effect, but would be restrictive to specific conditions (such as preventive breast cancer screening) that are likely to be insensitive approximations.

Fairchild and McDaniel (2017) pointed out that mediation is mostly appropriate in data contexts where temporality can be established. They argued that examining mediation analyses with cross-sectional data requires the researcher to provide a compelling rationale that temporal ordering of the examined variables is correct. Income must precede a biological stress response. Ideally, income would be measured at time *T*−1. However, stress and income levels are expected to exist concurrently. As the psychosocial stress hypothesis de-emphasizes material well-being for the lived experience of relative income, it should result in temporal overlap. Given that ESS data are repeated cross-sections and not repeated individual observations, no before-and-after treatment may be observed at the individual level. In this study, direct, total, and mediated effects should be understood as correlational in nature. Mediation being identified in data is not the same as concluding a process of mediation. However, mediation as a process linking income to health is theoretically plausible. This study primarily infers on the likelihood of these causal pathways.

Still, the possibility that the income-health relationship is reversed or bi-directional is a fundamental issue in cross-sectional studies. Ill-health may impact the probability of employment, and experiencing a health shock increases the likelihood of leaving employment and transition into disability (García-Gómez, 2011). Early life health conditions may constrain economic success in adulthood, as ill-health in childhood may affect opportunities to acquire education or reduce the efficiency of schooling (O’Donnell et al., 2015). Psychosocial theory attempt to create a link between socioeconomic positioning and health outcomes by directing attention to endogenous biological responses to human interaction (Krieger, 2001). Extending the health selection argument, it is possible that ill-health causes psychosocial stress for instance through difficulties with coping or onset depression. However, it is difficult to conceive of reverse *psychosocial mediation* from health to income in this case; the direct mechanisms from ill-health to reduced income seem more likely.

This study considers age as a confounder of the income–health relationship and is agnostic to age-differentiated causal mechanisms between income and health. It also includes respondents ranging from adolescence to old age. Earlier research has suggested that the relationship between income and health varies over the life-course. This is particularly apparent in age groups where transitioning between age-stratified institutions are common; labor market entry and retirement ages (Rehnberg et al., 2021). This age-differentiated relationship extends to age-specific causal mechanisms. Hoffmann et al. (2018) argue that social causation is more important than health selection in the second part of the life course, in the transition from adulthood to old age. While this study does not address age-specific mechanisms, including all age-groups available in the statistical model aligns with the universal assumption in psychosocial theory; that perceptions of relative positioning in the social hierarchy are always present and that all citizens are to some extent subject to the hypothesized effects of income inequality (Lynch et al., 2000).

A natural extension for future research includes comparative analyses of repeat observations from individuals in order to investigate to what extent changes in individual income or psychosocial stress affect health outcomes and changes in health outcomes differently, depending on economic context. Future studies may also attempt to parse the mediative effect of psychosocial stress on the income–health relationship by age-groups, in order to specify the exact mechanisms at play at different stages of the life-course.

## 7 Concluding remarks

Individual-level mechanisms remain important for explaining the income–health gradient in Europe. Evidence of the IIH is mixed, and the psychosocial stress mechanism should be pursued and researched further insofar as it may represent a biological response to individual income levels. While effects of individual income remain relevant, the effects of income are not merely material; a higher level of material comfort tends to correlate with a lower level of psychosocial stress.

Lacking evidence of an income inequality effect specifically does not entitle policymakers to avoid redistributive income policies. Policies should be aimed at allowing a greater number of people to live with a certain degree of material comfort and a reduced sense of financial precarity. Reducing income inequality by targeting those at a comparatively low income, reducing the potential consequences facing low income earners through generous welfare benefits, and ensuring an equitable distribution of public and private resources remain potential pathways to achieve health gains through both material and psychosocial mechanisms, despite the lack of convincing evidence for the IIH specifically.

## Declaration of competing interest

None.

## Figures and Tables

**Figure A1:**
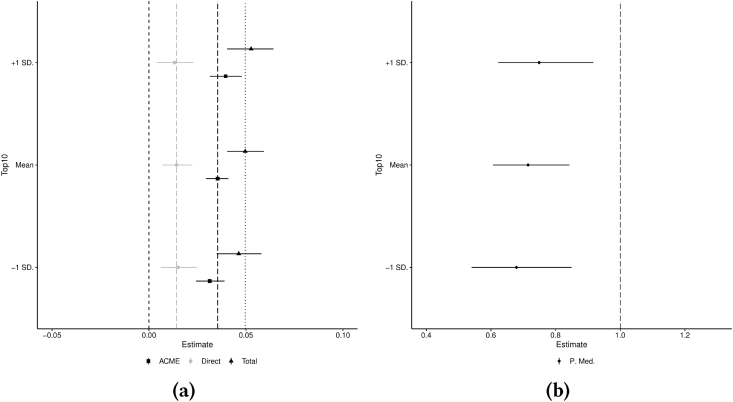
Left: ACME, direct, and total effects of multilevel moderated mediation model at three different levels of income inequality (top 10% income share). Right: Proportion of mediated effect at three levels of income inequality.

**Figure A2:**
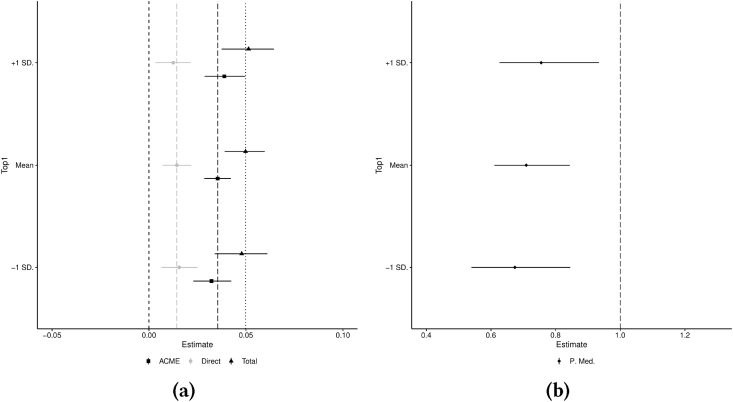
Left: ACME, direct, and total effects of multilevel moderated mediation model at three different levels of income inequality (top 1% income share). Right: Proportion of mediated effect at three levels of income inequality.

**Figure A3:**
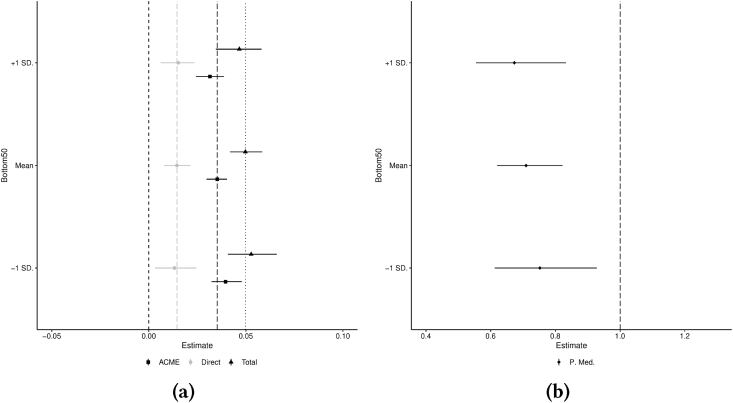
Left: ACME, direct, and total effects of multilevel moderated mediation model at three different levels of income inequality (bottom 50% income share). Right: Proportion of mediated effect at three levels of income inequality.

**Figure B2:**
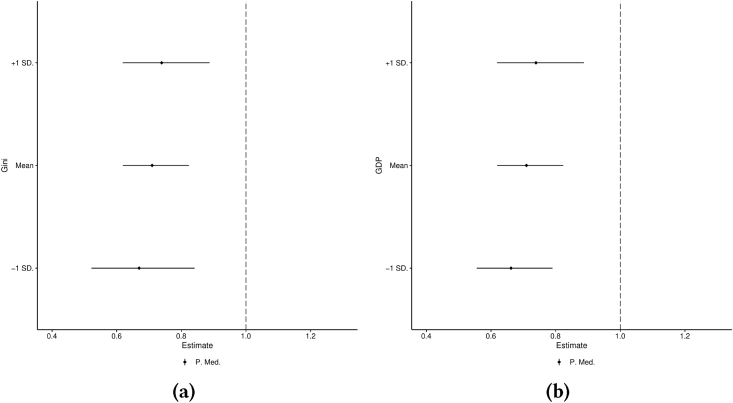
Left: Proportion of mediated effect of income from multilevel moderated mediation model at three different levels of income inequality. Right: Proportion of mediated effect of income from multilevel mediation model at three different levels of GDP per capita. Both figures include 95% bootstrap confidence intervals. Both models based on N = 28814 observations by listwise deletion.

**Table C1:** List of indicators, anchor labels, and transformation output for all categories in all items used in the psychosocial stress index. Note that ampersands and hyphenation indicate separate categories and ranges on the original scale. Items 2–9 share anchor labels and were separated in table to show the inverted collapsed scale.

**#**	**Item**	**Original**	**Collapsed**
**1**	Feelings about income	Living comfortably	0
		Coping	1
		Difficult	2
		Very difficult	3
**2**–**7**	Felt depressed	None or almost none of the time	0
	Effort, how often	Some of the time	1
	Feel sad, how often	Most of the time	2
	Can't get going, how often	All or almost all of the time	3
	Sleep was restless		
	Lonely, how often		
**8**–**9**	Happy, how often	None or almost none of the time	3
	Enjoying life, how often	Some of the time	2
		Most of the time	1
		All or almost all of the time	0
**10**–**11**	Influence work policy	Had no influence	3
	Autonomy at work	1–4	2
		5–9	1
		Had complete control	0
**12**	Meet friends, how often	Never & Less than once a month	3
		Once a month & Several times a month	2
		Once a week	1
		Several times a week & Every day	0
**13**	Intimate relationships	None	3
		1–3 & 4-6	2
		7–9	1
		10 or more	0
**14**	Social activities, how often	Much less than most	3
		Less than most	2
		About the same & More than most	1
		Much more than most	0

## Data Availability

Data will be made available on request.
